# Mendel: From genes to genome

**DOI:** 10.1093/plphys/kiac424

**Published:** 2022-09-12

**Authors:** Frances C Sussmilch, John J Ross, James B Reid

**Affiliations:** Discipline of Biological Sciences, School of Natural Sciences, University of Tasmania, Sandy Bay, Tasmania 7005, Australia; Discipline of Biological Sciences, School of Natural Sciences, University of Tasmania, Sandy Bay, Tasmania 7005, Australia; Discipline of Biological Sciences, School of Natural Sciences, University of Tasmania, Sandy Bay, Tasmania 7005, Australia

## Abstract

Two hundred years after the birth of Gregor Mendel, it is an appropriate time to reflect on recent developments in the discipline of genetics, particularly advances relating to the prescient friar’s model species, the garden pea (*Pisum sativum* L.). Mendel’s study of seven characteristics established the laws of segregation and independent assortment. The genes underlying four of Mendel’s loci (*A*, *LE*, *I*, and *R*) have been characterized at the molecular level for over a decade. However, the three remaining genes, influencing pod color (*GP*), pod form (*V/P*), and the position of flowers (*FA/FAS*), have remained elusive for a variety of reasons, including a lack of detail regarding the loci with which Mendel worked. Here, we discuss potential candidate genes for these characteristics, in light of recent advances in the genetic resources for pea. These advances, including the pea genome sequence and reverse-genetics techniques, have revitalized pea as an excellent model species for physiological–genetic studies. We also discuss the issues that have been raised with Mendel’s results, such as the recent controversy regarding the discrete nature of the characters that Mendel chose and the perceived overly-good fit of his segregations to his hypotheses. We also consider the relevance of these controversies to his lasting contribution. Finally, we discuss the use of Mendel’s classical results to teach and enthuse future generations of geneticists, not only regarding the core principles of the discipline, but also its history and the role of hypothesis testing.

## Teaser

The molecular nature of Mendel’s genes, the genetic resources available for pea, pea as a model for teaching and research, and recent controversies about Mendel’s data are discussed.

## Introduction

This year marks the 200th anniversary of Gregor Mendel’s birth—July 20, 1822. It is, therefore, timely to reflect on and revisit recent advances in genetics research using his model species, the garden pea (*Pisum sativum*). In 2011, two reviews discussed progress toward the molecular characterization of Mendel’s seven genes (Ellis et al., 2011; Reid and Ross, 2011). Now we report on the intervening 11 years, a period that has seen exciting developments relating to Mendel’s work and to pea genetics in general. Recent breakthroughs have reinvigorated the early tradition of pea as a model species for physiological–genetic studies (from the 1950s to the 1970s), which stemmed originally from the fact that some of Mendel’s genes control traits of paramount physiological and agronomic importance. An early example was Barber et al. (1958), who studied the effects of mutations in Mendel’s *LE* gene on flowering and internode length in pea, using genetics to further understand the physiology of these traits. Key discoveries in pea have been made recently on phenomena such as the regulation of flowering, nodulation, hormone biosynthesis and signaling, branching, and starch production (e.g. Barbier et al., 2019; Meitzel et al., 2021; Velandia et al., 2022; Williams et al., 2022). Pea also continues to attract attention as an important temperate crop, due to its ability to fix nitrogen and the high quality and quantity of protein in its seeds for both human and animal consumption. Further, there is great potential to breed pea varieties for improved yield and phosphorus use efficiency (Foyer et al., 2016; Powers and Thavarajah, 2019; Davies and Muehlbauer, 2020).

ADVANCES

- Four of Mendel’s seven genes have been characterized and we can now propose candidates for the three remaining genes.
- There have been major developments in the resources available for pea genetics, including sequence information and reverse genetics techniques.
- The controversies surrounding Mendel’s data can perhaps now be concluded.
- Technological advances have reinvigorated pea as a model for physiological genetics.
- Pea continues to facilitate discoveries in important aspects of plant biology.

Recent papers including those by van Dijk et al. (2022), Fairbanks (2022), Nasmyth (2022), and Berger (2022) discuss issues such as the history behind Mendel’s approach to his experiments, his biographical details, the nature of the questions he investigated, and how his studies relate to modern-day research fields. Here, we update our current understanding of Mendel’s genes, review the molecular progress that has occurred since 2011, and discuss how some of these advances have been used in a physiological context (see “Advances”). Other aspects reviewed here include recent criticisms of Mendel’s data and the use of Mendel’s genes as a foundation for the teaching of genetic principles.

## Molecular characterization of four of Mendel’s genes

The characterization of Mendel’s genes at the *R*, *LE*, *I*, and *A* loci occurred with the development of molecular techniques between 1990 and 2010. These results have been reviewed by Ellis et al. (2011) and Reid and Ross (2011), and also by Smýkal (2014), so only a brief summary will be given here. The identification of these genes was aided by the simple fact that in all four cases there was little dispute about which locus was responsible for the Mendelian characteristic concerned. In addition, all four characteristics are important in commercial cultivars of pea, giving these loci higher priority.

### Seed shape character—Round (*R*) versus wrinkled (*r*)

*R* was the first of Mendel’s seven genes to be characterized at a molecular level. It was shown to be involved in starch biosynthesis, encoding one of the major isoforms of starch-branching enzyme 1 (PsSBE1; Bhattacharyya et al., 1990). The nature of the *r* mutation used by Mendel seems clear, and results from a 0.8-kb insertion of a nonautonomous type II transposon. This mutation can explain the complex seed phenotype of *r* seeds, which—in addition to the wrinkled phenotype reported by Mendel—includes the shape of starch grains in the cotyledons (Gregory, 1903) and an elevated content of the sugars sucrose, fructose, and glucose (Stickland and Wilson, 1983).

### Stem length—Tall (*LE*) versus dwarf (*le*)

The difference at the *LE* locus appears to have been used in agriculture for over 500 years (Blixt, 1972), with the short-internode dwarf (*le-1*) types resulting in reduced stem elongation and hence reduced lodging and consequential disease susceptibility. The *LE* gene was shown to encode a gibberellin 3-oxidase (PsGA3ox1) that activates the inactive precursor GA_20_ to the biologically active GA_1_ (Lester et al., 1997; Martin et al., 1997). Along with results from a null mutant, *le-2*, this confirmed that 3-oxidation is necessary for activation of gibberellins, a key group of plant hormones (Lester et al., 1999). The mutant allele *le-1* used by Mendel is caused by a single base G-to-A mutation that results in an alanine to threonine substitution near the active site of the enzyme, reducing, but not eliminating, its activity (Ingram et al., 1984; Lester et al., 1997, 1999). Subsequent research identified the second member of the pea GA 3-oxidase family, which appears to compensate for the lack of *PsGA3ox1*(*LE*) activity in the seeds and roots of the *le-1* mutant, enabling this mutation to be used in agricultural cultivars (Weston et al., 2008).

### Cotyledon color—yellow (*I*) versus green (*i*)

The green coloration of the cotyledons observed by Mendel, as opposed to yellow cotyledons in wild-type plants, is due to a mutation in a *STAY-GREEN* (*SGR*) gene (Armstead et al., 2007; Sato et al., 2007). Similar “stay-green” mutants had previously been described in other species and seemed to be due to a reduction in the breakdown of chlorophyll during dark incubation, a phenotype also present in the leaves of pea *i* mutants (Armstead et al., 2007; Jiang et al., 2007; Sato et al., 2007; Aubry et al., 2008). Even after molecular characterization of *I*/*SGR* (Sato et al., 2007), the actual function of the gene remained unclear (Aubry et al., 2008; Sato et al., 2009), as was the mutation that Mendel actually used (Sato et al., 2007). Subsequently, stay-green mutants have been studied closely in diverse species over the last decade because of their potential to prolong photosynthesis and to increase yield in breeding programs. Findings from these studies indicate that Mendel’s *I* (*SGR*) gene acts in chlorophyll *a* degradation as a Mg-dechelatase (Shimoda et al., 2016; Jiao et al., 2020).

### Seed coat/flower color—purple (*A*) versus white (*a*)

Mendel (1866) noted that colored seed coats (testas), colored flowers, and pigmented leaf axils always occurred together. This indicates that they are pleiotropic characters caused by a single gene. *A* has since been shown to be a regulatory gene coding for a basic helix–loop–helix transcription factor that controls the spatial expression of the chalcone synthase gene family, essential for general flavonoid biosynthesis (Statham et al., 1972; Harker et al., 1990; Hellens et al., 2010). Mendel probably used an *a* allele caused by a G-to-A mutation at a splice site that results in a frameshift and premature stop codon (Hellens et al., 2010).

## Progress on the molecular characterization of Mendel’s three remaining genes

**Figure 1:**
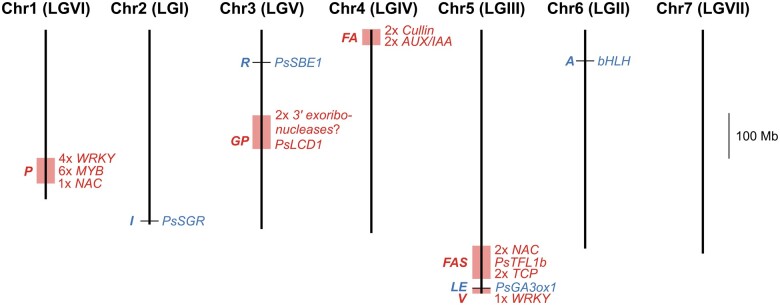
Mendel’s characterized genes and candidates. Vertical lines represent pea chromosomes, with corresponding linkage groups indicated (Kreplak et al., 2019). Mendel’s loci are indicated on the left-hand side, with characterized genes in blue and potential candidate genes in red on the right-hand side. See Supplemental Table S1 for more details.

Of the three remaining characteristics described by Mendel, “pod color” has two candidate genes awaiting final confirmation of causality, but “pod form” and “position of flowers” are complicated by a lack of clarity over which one of multiple possible loci associated with these traits was segregating in Mendel’s crosses. In earlier studies, the relative location of these loci was denoted across pea’s seven linkage groups (LGI-VII), based on the frequency of co-inheritance with morphological and/or molecular markers. When *Medicago truncatula* genome resources first became available (Cannon et al., 2006; Young et al., 2011), pea geneticists took advantage of the close synteny between *Medicago* and pea (e.g. Bordat et al., 2011) to identify candidate genes for pea loci of interest. Then, in a major development, Kreplak et al. (2019) published the pea genome itself. This now enables candidate genes for pea loci to be directly identified in the corresponding region of the pea genome (Figure 1), with the caveat that the current assembly represents ∼88% of the full genome and that some sequence scaffolds have not yet been assigned to a chromosome (Kreplak et al., 2019).

### Pod color—green (*GP*) versus yellow (*gp*)

**Figure 2:**
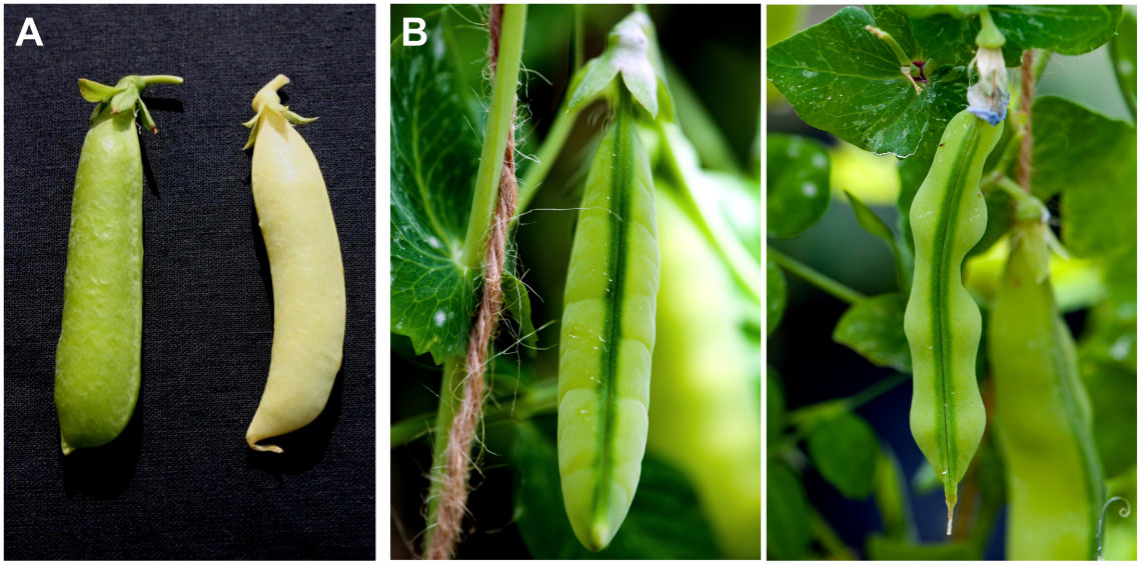
Mendel’s pod color and form characters. A, Pod color—green/*GP* (left), yellow/*gp* (right). B, Pod form—inflated, with sclerenchymatous tissue (left), constricted without sclerenchymatous tissue (right). Photographs provided by Prof. Wojciech Święcicki (A) and Dr. Robert Wiltshire (B).

When Mendel (1866) observed segregation of pod color, he found the allele for green pods dominant over that for yellow (Figure 2A; the opposite of cotyledon color, where green coloration is recessive). Yellow-podded (*gp*) plants have ˂5% of the chlorophyll levels in the pod mesocarp compared with wild-type plants (Price et al., 1988). The pod color locus (*GP*) was mapped to a region later found to correspond to pea Chromosome 3 in the reference genome assembly (Figure 1 and Supplemental Table S1; Lamprecht, 1948; Kreplak et al., 2019).

Recently, Shirasawa et al. (2021) identified two potential candidates for the pod color gene, through whole-genome resequencing of a yellow-podded pea variety (JI128), combined with genome-wide association study (GWAS) and transcriptome analysis-based approaches. They confirmed and narrowed the genetic map position of *GP*, but their GWAS data indicated association with sequences that have not yet been assigned to chromosomes in the pea genome. They found one gene, predicted to encode a 3′-exoribonuclease (Psat0s4355g0080), to contain a single-nucleotide polymorphism (SNP) that would result in a substitution (threonine to lysine) between green and yellow-podded lines. Shirasawa et al. (2021) also noted that an adjacent gene encoded another 3′ exoribonuclease, with SNPs and indels in the predicted promoter region and higher expression in the yellow-podded line. However, further research is needed to confirm if either of these candidate genes underlies Mendel’s pod color locus. In other species, 3′ exoribonucleases are known for their role in mRNA degradation (e.g. Nguyen et al., 2015) including in cell death initiation (Xi et al., 2009); any mechanism for these proteins in modifying chlorophyll content remains to be characterized.

Previously, Ellis et al. (2011) noted the presence of a gene similar to *LOWER CELL DENSITY 1* (*LCD1*) from Arabidopsis (*Arabidopsis thaliana*) in the region of the *Medicago* genome syntenic to the *GP* locus on pea LGV, and suggested the corresponding pea gene as a putative candidate for *GP*. Arabidopsis *lcd1* mutant plants show a pale phenotype with reduced chlorophyll content (Barth and Conklin, 2003), comparable to the chlorophyll deficiency seen in pods of pea *gp* plants. Now that the genomes for green (*GP*) and yellow-podded (*gp*) varieties of pea are available (Kreplak et al., 2019; Shirasawa et al., 2021), we can confirm both that there is a putative ortholog of *LCD1* in the corresponding region of pea Chromosome 3 (Figure 1) and that there is a SNP that would result in the substitution of a highly conserved, nonpolar isoleucine residue within the transmembrane domain with a polar threonine in the available sequence for the *gp* line JI128 (see Supplemental Table S1 for sequence details). Given the precedence for *LCD1* genes influencing chlorophyll levels, and the presence of a sequence difference between *GP* and *gp* that may affect protein function, this gene remains a strong candidate for *GP*.

### Pod form—inflated (*V*/*P*) versus constricted (*v*/*p*)

Mendel (1866) observed differences in the form of ripe pods with WT “inflated” pods that have a “parchment layer” on the inside of the pod wall comprising lignified cells/sclerenchyma, dominant over the recessive “constricted” form in which this layer is lacking/incomplete. The constricted pods are consequently wrinkled and deeply constricted between the seeds (Figure 2B), and are edible while immature, leading to them being known as “sugar” pods. In addition, the absence of a parchment layer is associated with pod indehiscence.

Two complementary loci controlling development of the parchment layer have been described in pea—*V* and *P* (White, 1917). It is not clear which of these would have been segregating in Mendel’s population, but early verbal comments suggest *V* was more likely (see Reid and Ross, 2011) and this is supported by a re-analysis of Mendel’s data by Ellis et al. (2019) and Weeden (2016), on the basis of phenotype. *V* has been mapped beneath *LE* on the genetic/linkage map (Weeden et al., 1993), a region that corresponds to the end of pea Chromosome 5 with around 200 annotated genes (Figure 1 and Supplemental Table S1; Kreplak et al., 2019). This region includes a gene encoding a WRKY transcription factor—a family previously linked to lignification (Wang et al., 2007; Guillaumie et al., 2009; Yang et al., 2016; Xie et al., 2021) and pod indehiscence/seed shattering (Tang et al., 2013; Parker et al., 2020). This gene could be investigated further as a candidate for *V*.

It is harder to match linkage information for *P* with a physical chromosomal location, as *P* is rarely included in linkage maps with molecular markers that can be matched to the pea genome. *P* has been mapped to a region of LGVI/pea Chromosome 1 (Weeden et al., 1998; Bordat et al., 2011; Kreplak et al., 2019) that contains genes encoding four WRKY transcription factors (see above), a NAC transcription factor homologous to genes that affect lignification in other legumes (Wang et al., 2011; Dong et al., 2014), and six MYB transcription factors homologous to genes that control lignification in other species (Figure 1 and Supplemental Table S1; Yang et al., 2007; Zhang et al., 2016; Gui et al., 2019). Narrowing down the chromosomal location of the *P* locus would help to reduce this list of potential candidates.

### Position of flowers—axial (*FA*/*FAS*) versus terminal (*fa*/*fas*)

The last of Mendel’s loci remaining to be characterized is the “position of flowers.” Mendel (1866) noted axial positioning of flowers (distributed at nodes along the main stem in the form of a compound raceme) was dominant over terminal positioning of flowers (all grouped together at the top of the stem, in the form of a “false umbel”). This condition is now known as fasciation, and involves the shoot apical meristem becoming elongated outward, perpendicular to normal acropetal stem growth, enabling multiple inflorescences to be borne from the top of the stem. Two loci affecting this characteristic have been described in pea, either of which may have been segregating in Mendel’s experiments: *FA* and *FAS* (see discussion by Ellis et al., 2011; Reid and Ross, 2011).

*FA* has been mapped to the top of LGIV (Laucou et al., 1998; Bordat et al., 2011), to a region corresponding to the start of pea Chromosome 4 (Kreplak et al., 2019). This region contains genes encoding (i) cullin family members—components of SCF ubiquitin ligase complexes involved in mediating auxin and jasmonic acid responses with mutant phenotypes including aberrant patterns of cell division or fasciation in other species (Shen et al., 2002; Stirnberg et al., 2002; Dohmann et al., 2005)—and (ii) auxin/indole-3-acetic acid (AUX/IAA) family members homologous to those linked to the regulation of meristem boundary domains and inflorescence architecture (Galli et al., 2015), which could be investigated further as candidates for *FA* (Figure 1 and Supplemental Table S1).

*FAS* has been mapped to LGIII but is not linked to *LE* (Sinjushin et al., 2006). This region corresponds to part of pea Chromosome 5 that includes a number of genes that are plausible candidates for *FAS* (Figure 1 and Supplemental Table S1). These include genes encoding transcription factors from the TCP family associated with branching/inflorescence development/floral organ morphogenesis and cell proliferation in other species (e.g. Cubas et al., 1999; Koyama et al., 2010; Kieffer et al., 2011), and the NAC family linked to fasciation in other species (Weir et al., 2004). In addition, a pea homolog of the Arabidopsis inflorescence meristem identity gene *TERMINAL FLOWERING 1*—*PsTFL1b*—is also a potential candidate in this region.

Homologs of the shoot apical meristem maintenance gene *CLAVATA1* (*CLV1*) have previously been suggested as potential candidates for *FA* and *FAS* (Ellis et al., 2011). We can now confirm that there are indeed pea members of the *CLV1/BARELY ANY MERISTEM (BAM)1/2/3* subfamily of leucine-rich repeat receptor-like protein kinase genes on pea Chromosomes 4 (*PsBAM1*) and 5 (*PsBAM3*), which may share redundancy with *CLV1* in meristem functioning similar to Arabidopsis *BAM* homologs (Nimchuk et al., 2015). However, integration of mapping (Laucou et al., 1998; Sinjushin et al., 2006) and genome information (Kreplak et al., 2019) indicates that both of these pea *BAM* genes fall outside the regions of immediate interest for *FA* and *FAS* (Supplemental Table S1).

## Progress with molecular tools and resources for pea as a model plant

When we last reflected on progress with characterizing Mendel’s loci 11 years ago (Reid and Ross, 2011), sequence data for pea were limited to some expressed sequence tag databases (see Bordat et al., 2011), with transcriptome information from next-generation sequencing just becoming available (e.g. Franssen et al., 2011). Molecular studies in pea commonly made use of the more comprehensive sequence resources available for other model legumes, including the closely related galegoid legume *M. truncatula* (Young et al., 2005, 2011), in addition to *Lotus japonicus* (Sato et al., 2008) and soybean (*Glycine max*; Schmutz et al., 2010). Functional characterization of gene phenotypic effects was often successfully achieved by a forward-genetics, candidate gene approach, with genetic mapping and identification of gene candidates using sequence resources for the closely syntenic *Medicago* (Kaló et al., 2004; Aubert et al., 2006; Bordat et al., 2011). Platforms for reverse genetics using Targeting-Induced Local Lesions IN Genomes (TILLING) had been developed for pea (Triques et al., 2007; Dalmais et al., 2008), and were also being successfully adopted in functional studies (e.g. Hofer et al., 2009).

In the past decade, we have seen first an increase in transcriptome data and improved genetic mapping of pea loci (e.g. Kaur et al., 2012; Duarte et al., 2014; Alves-Carvalho et al., 2015; Tayeh et al., 2015), then a chromosome-level assembly of the genome (Kreplak et al., 2019), and more recently genome sequences for additional pea lines (Shirasawa et al., 2021), paving the way for future pangenomics studies to compare core and variable gene sets between pea cultivars. At the same time, rapid expansion of genomic resources for other species has advanced comparative genomics between diverse legumes (e.g. Varshney et al., 2013; Schmutz et al., 2014; Griesmann et al., 2018; Zhuang et al., 2019; Quilbé et al., 2021). New reverse genetics tools are also being developed, with a recent report of successful gene-editing via optimization of the clustered regularly interspaced short palindromic repeats (CRISPR)/CRISPR-associated protein 9 (Cas9) system in pea (Li et al., 2022). While transformation in pea is still problematic, it is being used extensively for transient expression in roots (e.g. Clemow et al., 2011) and even stable expression, including for one of Mendel’s genes, *LE* (Reinecke et al., 2013) and a gene involved in seed filling (Meitzel et al., 2021).

These recent molecular advances have facilitated major physiological advances in pea over the last decade. For example, the reverse genetics approach, TILLING, allowed Tivendale et al. (2012) to confirm that tryptophan is converted to the key plant hormone auxin by just two steps, as in Arabidopsis (Mashiguchi et al., 2011). Despite the importance of auxin for plant growth and development, its biosynthetic pathway had remained elusive for decades. Next it was demonstrated, by disrupting auxin biosynthesis in maturing pea seeds, that auxin is required for normal starch synthesis (McAdam et al., 2017). This exciting demonstration of the auxin–starch relationship has since been confirmed for the seeds of maize (*Zea mays*; Bernardi et al., 2019) and rice (*Oryza sativa*; Zhang et al., 2021). Auxin also provides an interesting indirect link between two of the great 19th Century plant biologists, Mendel and Charles Darwin. The discovery of auxin emanated (after many decades) from Darwin’s studies on phototropism (Darwin and Darwin, 1896). Now we know that auxin affects starch biosynthesis, as does Mendel’s *R* gene. Furthermore, Mendel’s *LE* gene turned out to be an auxin-regulated gene (Ross et al., 2000; O’Neill et al., 2010).

Other examples where pea genes have provided valuable insights into important physiological processes include the identification of the branching hormone, strigolactone (Gomez-Roldan et al., 2008), and its associated biosynthetic and perception pathways (see Mashiguchi et al., 2021). Pea is also proving to be a key model species for genetic studies on plant–microbe interactions during symbioses such as nodulation and arbuscular mycorrhizae (Foo et al., 2013; Velandia et al., 2022), phenomena that do not occur in Arabidopsis.

## Mendel’s data

### Did Mendel’s characters involve discrete differences?

For Mendel’s research on the seven characters he selected, segregation into just two forms was an essential property. However, on the rediscovery of Mendel’s findings, this binary classification was soon questioned, mainly by Weldon (1902). Subsequent generations of geneticists (1910–2010) accepted that in F_2_ segregations, Mendel’s characteristics are typically binary, as demonstrated by data sets collated in Weeden (2016). Nevertheless, Weldon’s ideas have been resurrected by Radick (2015), who again posed the question of “whether Mendel was right to work with just the two categories in the first place.” A photograph of pea seeds presented by Weldon (1902) showing seeds varying from yellow to green in a continuous manner, rather than falling into clear yellow and green categories as described by Mendel (1866), has been reproduced in support of these claims (Radick, 2015; Arney, 2019). When extrapolated to F_2_ generations, Weldon (1902) and Radick (2015, 2022) appear to challenge the binary nature of segregations. This is a much more fundamental challenge to Mendelian genetics than simply pointing out, as Weeden (2016) appears to, that for some characteristics there can be a minority of “ambiguous” segregates in between two otherwise distinct and larger groups.

**Figure 3:**
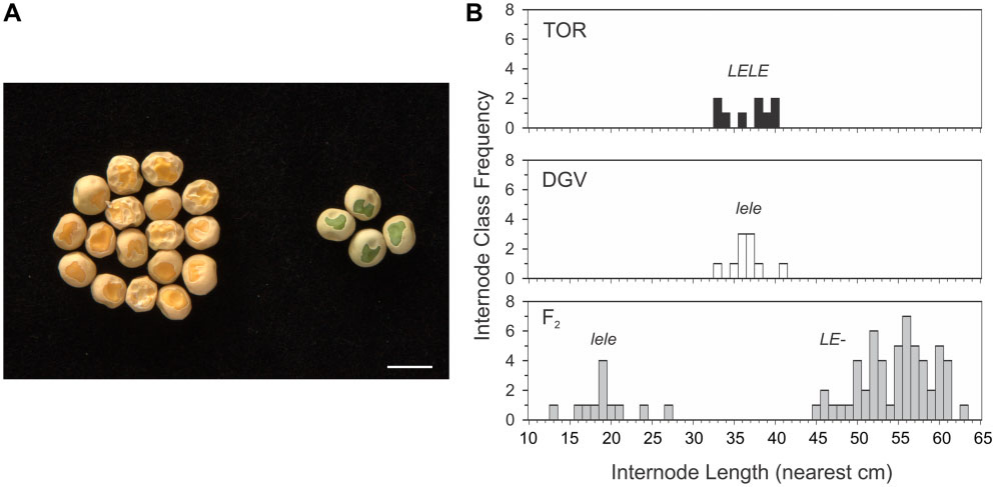
Clear segregation of cotyledon color (*I*/*i*) and stem length (*LE*/*le*). A, F_2_ seeds from a cross between lines Torsdag (*II*) and 53 (*ii*), showing segregation of cotyledon color (scale bar = 1 cm). The round/wrinkled (*R*/*r*) difference is also segregating. B, Stem length data from Torsdag (TOR, wild-type, tall, *LELE*), Dippes gelbe Viktoria (DGV, dwarf, *lele*), and the F_2_ generation of a cross between these lines, showing segregation of the tall/dwarf difference from data in Ross and Reid (1987).

We refute this challenge, by showing here that characters such as cotyledon color can be classified into discrete groups. In our collection of pea genotypes, we have lines with 100% yellow cotyledons or 100% green cotyledons. When these lines are crossed, the F_2_ seeds clearly fall into the yellow or green categories, provided that the seed coat is partially removed to expose the cotyledons (Figure 3A), as also done by Weldon (1902). Clear segregation is also apparent in a photo modified by van Dijk et al. (2022), originally from Darbishire (1911). Further supporting our view, Mendel’s tall/dwarf difference also segregates cleanly in the F_2_, even if the parents are overlapping in height because of other genetic factors. In a cross between one of the shortest tall lines and one of the largest dwarf lines in our collection, we found the tall (*LE-*) and dwarf (*lele*) plants to be easily recognizable (Figure 3B). These examples show that it is possible to obtain pure parental lines that are consistent for Mendel’s characters, and which produce discrete bimodal segregations in the F_2_ generation (Figure 3), even when the parental phenotypes overlap. Therefore, the likelihood that Mendel observed clear F_2_ segregations appears beyond dispute, although we cannot exclude the possibility that some of his segregations did contain individuals with “ambiguous” phenotypes.

At the beginning of the last century, Weldon (1902) was perhaps justified to question how Mendel’s differences relate to the total variation that can be observed for each character. At present, however, geneticists understand that the expression of some of Mendel’s characters can be influenced by other genes and the environment. Bearing that in mind, we contend that the resurrection of Weldon’s ideas, largely from an historical perspective (Radick, 2015, 2022), has occurred with inadequate scrutiny of how his conclusions were reached or analysis of readily available data.

Mendel (1866) did examine, in a preliminary manner, certain traits that did not show binary segregation patterns. For example, he commented that time of flowering was not amenable to his analysis because the flowering time of hybrids stood almost exactly between the times of the two parents. By being selective in this way, Mendel concentrated on characteristics that enabled him to discover the laws of inheritance. Then, those laws could be, and indeed have been, extrapolated to apply to characters beyond those he originally described. This point seems to have been lost on some critics, who suggest that Mendel’s laws apply only to his carefully selected traits, and not to genetic variation in general (e.g. Arney, 2019).

Since Mendel’s time, however, clear segregations have been observed for the flowering time trait. Through the use of appropriate environmental conditions (short photoperiods and nonvernalizing temperatures), Murfet (1971) showed clear segregation between wild-type and mutant forms, for two flowering genes. After further detailed analysis, over 10 loci controlling flowering with Mendelian patterns of inheritance were identified without knowledge of their molecular nature or biochemical function (Reid et al., 1996). Since then, a range of molecular tools have been employed to identify most of these loci, in addition to other flowering genes as well. Candidate gene and comparative genetic approaches using knowledge and sequence information from other plant species initially proved highly effective for characterizing pea flowering genes/loci (see Hecht et al., 2005; Weller et al., 2009); and progress has been more rapid as legume genome and specific pea sequence resources became available (see Weller and Ortega, 2015; Williams et al., 2022). Thus, although flowering time is a quantitative trait, sensitive to environmental cues including photoperiod, a number of key genes have been identified based on their Mendelian patterns of inheritance.

### Mendel’s ratios

Mendel’s data have probably been examined and re-analyzed more than any other data in biology. This, by itself, highlights the importance of his work. The scientific method has been put to use in testing the theory that Mendel’s results agreed too closely with expectation—that is, his data were “too good.” Such questions arose with the re-discovery of Mendel’s work at the beginning of the 20th century (Weldon, 1902) and became widely debated in the literature after analysis by the eminent statistician (Fisher, 1936). Fisher (1936) concluded that “the data of most, if not all, of the experiments have been falsified so as to agree closely with Mendel’s expectations.” This was followed by comments by key evolutionary geneticists such as Wright (1966) and Dobzhansky (1967). The debate continued, with several publications during the 2000s (e.g. Hartl and Fairbanks, 2007; Franklin et al., 2008; Pires and Branco, 2010).

In the last decade, leading pea geneticists have entered the fray, on opposite sides (Weeden, 2016; Ellis et al., 2019). Weeden (2016) clearly agrees with the earlier suggestions that Mendel’s data were too good, collating those data and noting their relatively low chi-squared values. Weeden (2016) based on suggestions by Sturtevant (1965) proposed four explanations for the unexpectedly close fit to the predicted numbers. The first of these hypotheses was that in pea an unusual meiotic mechanism somehow results in that close fit. Weeden (2016) empirically refuted that possibility, by collating data from several pea geneticists, published since 1927. These data show that, in general, pea does not produce offspring ratios with a better fit than is expected. The second explanation was that Mendel excluded some data from his publication. Weeden (2016) accepted that possibility but considered the omissions inadequate in scale to explain the closeness of fit. The third explanation, supported by Weeden (2016), was that there may have been some bias in scoring ambiguous phenotypes. He presented statistical evidence that the closeness of fit “problem” is more obvious with characters prone (according to Weeden, 2016) to ambiguity, and hence to a biased classification. The fourth possibility was that “a portion” of Mendel’s data may have been falsified by an assistant or assistants. In summary, Weeden (2016) favored explanations three and four and did not exclude either.

Weeden (2016) also questioned “the lack of any statistically significant deviation in Mendel’s data from the expected ratios.” However, as noted by Ellis et al. (2019), Mendel did in fact report some substantial deviations from expectation. For example, one of his F_1_ plants produced 43 round and 2 wrinkled seeds, while another gave 14 round and 15 wrinkled (Mendel, 1866); neither set fits the expected 3:1 ratio. Mendel appears to have added these numbers into a grand total for that character, but clearly, not all of his data were “too good.”

When Ellis et al. (2019) subdivided some of the grand totals, sometimes into the offspring of individual plants, larger chi-squared values were often obtained, compared with those of the totals. An example is provided by the ratios between heterozygotes and homozygous dominants in F_2_ generations, as revealed by growing the F_3_ generation. In some cases, the expected ratio was 2:1, but in generations where only 10 F_3_ offspring from each F_2_ plant were tested, it should have been 1.8874:1.1126 (Fisher, 1936). Taking this into account, the mean chi-squared value for 27 F_2_ to F_3_ genotyping comparisons is 0.90, close to the expected value of 1 (data from Additional file 1: Table S1.3 of Ellis et al., 2019).

In summary, while Weeden (2016) essentially agrees with the previous criticism that Mendel’s data were too close to expectation, Ellis et al. (2019) strongly disagree, concluding that “there is nothing remarkable about Mendel’s data.” At the same time, of course, Weeden’s (2016) collated data from other published pea geneticists actually support Mendel’s laws. The current argument, therefore, is not about the correctness of those laws, but about whether or not Mendel’s data were too close to expectation. Interestingly, van Dijk et al. (2022) alluded to yet another controversy, this time regarding whether Mendel’s approach was essentially deductive or inductive. We note here that according to his 1866 paper, Mendel was certainly aware of, and indeed employed, the scientific method of defining hypotheses and then testing them (a deductive approach). In fact, it has been suggested that Mendel’s mastery of the scientific method was “far ahead of his time” (Huminiecki, 2020). This mastery was complemented by Mendel’s careful selection of pea as his major experimental material, ensuring he had true breeding lines at the commencement of his crosses, and his practice of observing actual numbers for each separate character over several generations after the initial cross. While individually these approaches had been used by earlier workers, Mendel’s combination of skills, due to his knowledge of both biology and mathematics, enabled him to make his discoveries.

Perhaps now the debates regarding Mendel’s approach and data can finally be put to rest, despite persisting for many decades. Mostly, they are peripheral to the essence of Mendel’s observations and to his invaluable insights into the inheritance of characteristics.

### Did Mendel miss linkage?

Related to discussions about Mendel’s data is the issue of how he missed the phenomenon of linkage. Prior to a major revision of the pea linkage map (Weeden et al., 1998), there was confusion, even in textbooks, about the location of Mendel’s seven genes. During the 1950s and 1960s, they were reported to be located on the seven different chromosomes of pea, although this was refuted as early as 1970 by Murfet and later by Blixt (1975). With our current knowledge we know that the *R* and *GP* loci are weakly linked (Weeden et al., 1998), which may not have been detected with the number of plants used by Mendel (Weeden, 2016), while if Mendel’s pod membrane gene was at the *V* locus, it is quite tightly linked to the stem length locus *LE* (Hall et al., 1997). It is worth emphasizing that Mendel did not note unusual frequency of co-inheritance of pod form and stem length characters in his segregating populations. It is possible that Mendel studied *V* in a population that was not segregating for both characters (Ellis et al., 2011), or that he may have instead studied pod form via segregation at the *P* locus, which is not linked to *LE*. This issue has been discussed in detail by Reid and Ross (2011) and Ellis et al. (2011). Whatever the reason for the lack of linkage detection by Mendel, it does not overshadow the brilliance of his insights into the inheritance of discrete characters, and the principles of segregation and independent assortment which are the foundations of the discipline of genetics.

## Mendel’s genes as a teaching tool

The story of Mendel’s experiments, their rediscovery after decades, and the controversies about his data that ensued, add to the benefits of garden pea as an excellent model for teaching, allowing students to appreciate the development of genetics as a discipline and the scientific method of hypothesis testing. A study of Mendel’s seven characteristics illustrates the main principles of genetics. These include the importance of dominant and recessive phenotypes, gene families, pleiotropy, structural and regulatory gene function, and the various methods available to identify genes. The mutations in Mendel’s genes include single base substitutions (*le-1*; Lester et al., 1997), disruption of splice sites (*a*; Hellens et al., 2010), and both small (*i*; Sato et al., 2007) and large insertions (*r*; Bhattacharyya et al., 1990). Students can be instructed in how Mendel’s genes have been pivotal for studying key aspects of plant development, including pigmentation patterns, seed development, the hormonal regulation of plant growth, and plant senescence. A genetics and plant development course can be based purely on these aspects. At the practical level, pea plants are easy to grow, and students can be introduced to the husbandry of commercially relevant plants bearing large flowers that self-pollinate but which can be easily crossed. Cultivars carrying Mendelian mutations (e.g. *le*, *a*, *r*, and *i*) are readily available at plant nurseries. Overall, students may not only learn the key principles of genetics and plant development, but also relate to the history of the discipline and the associated controversies about Mendel’s data.

## Conclusion

In the last decade, Mendel’s results have been reviewed by pea geneticists including Weeden (2016), Ellis et al. (2019), and van Dijk et al. (2022). Weeden (2016) notes that “whether Mendel should be placed on a pedestal as the founder of experimental genetics is still a moot point.” In contrast, Ellis et al. (2019) describe Mendel’s (1866) paper as “exemplary,” and its subsequent statistical criticism as a “pernicious feature.” Within the recent reviews, a difference of opinion also emerges with regard to the possibility of ambiguous phenotypes interfering with the scoring of Mendel’s characters. According to Weeden (2016), ambiguous individuals can occur with regard to four of Mendel’s characters, including cotyledon color and seed shape. In contrast, van Dijk et al. (2022) recently gave the impression that in general terms the segregation of cotyledon color and seed shape “in the F_2_ is very obvious.” Here, we agree that segregation for Mendel’s characters can indeed be unambiguous (Figure 3). We also dismiss recent doubts about the fundamentally binary nature of Mendel’s characters (Radick, 2015). However, at the same time, we should not deny the existence of ambiguous individuals in some circumstances (Weeden, 2016). It is clear that even if Mendel encountered some ambiguity at times, he would have observed more than enough clear segregations to form the basis for his laws.

OUTSTANDING QUESTIONS

- Will recent advances in molecular techniques allow us to identify Mendel’s three unknown genes?
- Is pod color (*GP*) controlled by a 3’ exoribonuclease gene, or is it still too soon to rule out another candidate?
- Have questions surrounding the validity of Mendel’s data finally been resolved?
- What does the future hold for pea genetics?

The title of our last review (Reid and Ross, 2011) was “Mendel’s genes: Toward a full molecular characterisation.” However, 11 years on, that full characterization is still yet to occur. One reason is that pea genes other than Mendel’s have often been prioritized for full characterization, based on their physiological or developmental importance. Another reason is that pea has not been an easy model species for molecular genetics research. In fact, with the expansion of plant molecular biology in the 1980s and 1990s, pea was quickly left in the wake of Arabidopsis, in which advances were facilitated by the relatively small genome and other features such as the paucity of repetitive sequences, ease of transformation, and rapid life cycle.

That situation is now changing, with pea catching up in some key molecular areas. Indeed, we now have the tools to complete the characterization of Mendel’s genes. On the basis of linkage/mapping studies, combined with the pea genome, candidate genes can readily be found. The reverse genetics techniques of TILLING and more recently CRISPR now provide mechanisms for obtaining mutants for these candidate genes, to compare the resulting phenotypes with those expected from Mendel’s descriptions. In addition to helping to identify the remainder of Mendel’s genes, the molecular advances will continue to benefit pea genetics in general (see “Outstanding Questions”). In fact, following recent speculation (Berger, 2022), we might surmise that Mendel would be happy to see those molecular advances propelling pea back to the top echelon of model plant species.

## Supplemental data

The following materials are available in the online version of this article.

**Supplemental Table S1.** Accession and chromosomal location details for Mendel’s genes and candidates identified for Mendel’s remaining loci shown in Figure 1.

## Supplementary Material

kiac424_Supplementary_Data

## Notes

All authors contributed ideas, drafted the manuscript, and revised the text. F.C.S. performed the bioinformatic analyses for candidate gene identification.

The author responsible for distribution of materials integral to the findings presented in this article in accordance with the policy described in the Instructions for Authors (https://academic.oup.com/plphys/pages/general-instructions) is: James Reid (jim.reid@utas.edu.au).
