# Supplementary material for: Mendel: From genes to genome
Source: Plant Physiol. 2022 Sep 12;190(4):2103–14. doi: 10.1093/plphys/kiac424 (PMC9706470; doi:10.1093/plphys/kiac424)
Supplement: kiac424_Supplementary_Data [file kiac424_supplementary_data.pdf]

Supplemental Table S1: Accession and chromosomal location details for Mendel's genes and candidates identified for Mendel's remaining loci shown in **Fig. 1**. Details based on *Pisum sativum* v1a Caméor ('Psat…') reference genome (Kreplak et al., 2019) or contig sequences (GenBank: 'BNEU…') of the yellow-podded *gp* line JI128 (Shirasawa et al., 2021) as indicated.

Chr/LG	Locus/gene/family name	Accession number	Chromosomal location	Reference
Chr1/LGVI	*WRKY* (*P* candidates)	Psat1g161840	310519245..310522209	This study
		Psat1g174040	325300509..325303432	
		Psat1g175040	326093926..326096427	
		Psat1g178960	330180323..330183676	
	MYB (*P* candidates)	Psat1g162600	311713809..311716053	This study
		Psat1g164560	314894320..314901952	
		Psat1g173600	324784784..324789948	
		Psat1g179560	330699011..330701374	
		Psat1g179600	330815840..330817704	
		Psat1g181000	332415497..332416690	
	NAC (*P* candidate)	Psat1g169200	319942921..319944895	This study
Chr2/LGI	*I/PsSGR*	Psat2g181040	419920198..419923651	Armstead et al. (2007); (Sato et al., 2007)
Chr3/LGV	*R/PsSBE1*	Psat3g034640	71664415..71704007	Bhattacharyya et al. (1990)
	Vc2 (marker ~15.6cM above *GP*)	Psat3g104920	207236465..207237116	Brauner et al. (2002)
	PsLCD1 (*GP* candidate)	Psat3g133000 (BNEU01008294)	259505436..259509443	This study
	Fbpase (marker ~2.3cM beneath *GP*)	Psat3g134680	261831660..261834245	Brauner et al. (2002)
	3' exoribonucleases (*GP* candidates)	Psat0s4355g0080	Scaffold not yet assigned to a pea chromosome but likely on PsChr 3 based on location of ortholog on syntenic Medicago Chr 7 (Medtr7g076910)	Shirasawa et al. (2021)
		Psat0s4355g0120		
Chr4/LGIV	*Cullin* (*FA* candidates)	Psat4g007120	8608415..8612231	This study
		Psat4g007920	9777612..9780545	
	Aux/IAA (*FA* candidates)	Psat4g012360	18212968..18214204	This study
		Psat4g020800	29668995..29669485	
	gdcL (marker ~10cM beneath *FA*)	Psat4g023520	33798841..33806207	Laucou et al. (1998)
	PsBAM1 (best Chr4 tBLASTn hit for AtCLV1)	Psat4g079880	134876825..134880270	This study; gene named by Smitha Ninan et al. (2017)

Chr/LG	Locus/gene/family name	Accession number	Chromosomal location	Reference
Chr5/LGIII	*PsBAM3* (best Chr5 tBLASTn hit for AtCLV1)	Psat5g195160	390754650..390758940	This study
	Pepcn (marker ~16.8cM above *FAS*)	Psat5g238360	474115077..474120235	Sinjushin et al. (2006)
	TCP (*FAS* candidates)	Psat5g240240	477780415..477781472	This study
		Psat5g274920	536962555..536964520	
	NAC (*FAS* candidates)	Psat5g245960	491537966..491540742	This study
		Psat5g277640	541653813..541654979	
	TFL1b (*FAS* candidate)	Psat5g256920	510686113..510687771	Foucher et al. (2003)
	PK4 (marker ~28.1cM beneath *FAS*)	Psat5g281640	546992970..547001760	Sinjushin et al. (2006)
	LE/*PsGA3ox1*	Psat5g299720	567365719..567368443	Lester et al. (1997)
	WRKY (*V* candidate)	Psat5g308000	578704614..578706779	This study
Chr6/LGII	*A*/*bHLH*	Psat6g060480	68330158..68340923	Hellens et al. (2010)

Supplemental References

Armstead I, Donnison I, Aubry S, Harper J, Hörtensteiner S, James C, Mani J, Moffet M, Ougham H, Roberts L, Thomas A, Weeden N, Thomas H, King I (2007) Cross-species identification of Mendel's *I* locus. Science **315:** 73-73

Bhattacharyya MK, Smith AM, Ellis THN, Hedley C, Martin C (1990) The wrinkled-seed character of pea described by Mendel is caused by a transposon-like insertion in a gene encoding starch-branching enzyme. Cell **60:** 115-122

Brauner S, Murphy RL, Walling JG, Przyborowski J, Weeden NF (2002) STS markers for comparative mapping in legumes. Journal of the American Society for Horticultural Science jashs **127:** 616-622

Foucher F, Morin J, Courtiade J, Cadioux S, Ellis N, Banfield MJ, Rameau C (2003) *DETERMINATE* and *LATE FLOWERING* are two *TERMINAL FLOWER1*/*CENTRORADIALIS* homologs that control two distinct phases of flowering initiation and development in pea. The Plant Cell **15:** 2742-2754

Hellens RP, Moreau C, Lin-Wang K, Schwinn KE, Thomson SJ, Fiers MWEJ, Frew TJ, Murray SR, Hofer JMI, Jacobs JME, Davies KM, Allan AC, Bendahmane A, Coyne CJ, Timmerman-Vaughan GM, Ellis THN (2010) Identification of Mendel's white flower character. PLOS ONE **5:** e13230

Kreplak J, Madoui M-A, Cápal P, Novák P, Labadie K, Aubert G, Bayer PE, Gali KK, Syme RA, Main D, Klein A, Bérard A, Vrbová I, Fournier C, d'Agata L, Belser C, Berrabah W, Toegelová H, Milec Z, Vrána J, Lee H, Kougbeadjo A, Térézol M, Huneau C, Turo CJ, Mohellibi N, Neumann P, Falque M, Gallardo K, McGee R, Tar'an B, Bendahmane A, Aury J-M, Batley J, Le Paslier M-C, Ellis N, Warkentin TD, Coyne CJ, Salse J, Edwards D, Lichtenzveig J, Macas J, Doležel J, Wincker P, Burstin J (2019) A reference genome for pea provides insight into legume genome evolution. Nature Genetics **51:** 1411-1422

Laucou V, Haurogné K, Ellis N, Rameau C (1998) Genetic mapping in pea. 1. RAPD-based genetic linkage map of Pisum sativum. Theoretical and Applied Genetics **97:** 905-915

Lester DR, Ross JJ, Davies PJ, Reid JB (1997) Mendel's stem length gene (*Le*) encodes a gibberellin 3 beta-hydroxylase. The Plant Cell **9:** 1435-1443

Sato Y, Morita R, Nishimura M, Yamaguchi H, Kusaba M (2007) Mendel's green cotyledon gene encodes a positive regulator of the chlorophyll-degrading pathway. Proceedings of the National Academy of Sciences **104:** 14169-14174

Shirasawa K, Sasaki K, Hirakawa H, Isobe S (2021) Genomic region associated with pod color variation in pea (*Pisum sativum*). G3: Genes, Genomes, Genetics **11:** jkab081

Sinjushin A, Konovalov F, Gostimskii S (2006) A gene for stem fasciation is localized on linkage group III. Pisum Genetics **38:** 19-20

Smitha Ninan A, Shah A, Song J, Jameson PE (2017) Differential gene expression in the meristem and during early fruit growth of *Pisum sativum* L. identifies potential targets for breeding. International Journal of Molecular Sciences **18:** 428
